# Citrate dose for continuous hemofiltration: effect on calcium and magnesium balance, parathormone and vitamin D status, a randomized controlled trial

**DOI:** 10.1186/s12882-021-02598-2

**Published:** 2021-12-11

**Authors:** Willem Boer, Tom Fivez, Margot Vander Laenen, Liesbeth Bruckers, Hans Jurgen Grön, Miet Schetz, Heleen Oudemans-van Straaten

**Affiliations:** 1 Department of Anesthesiology, Intensive Care Medicine, Emergency Medicine & Pain Medicine, Ziekenhuis Oost Limburg ZOL, Genk, Belgium; 2 I-BioStat, Data Science Institute, Hasselt University, Hasselt, Belgium; 3 Immundiagnostik AG, Bensheim, Germany; 4 Department of Laboratory and Intensive Care Medicine, Catholic University Leuven, Leuven, Belgium; 5 Amsterdam University Medical Centre, location VUmc, Amsterdam, the Netherlands

**Keywords:** Citrate, CVVH, Calcium, Magnesium balance, iPTH, oxPTH, noxPTH

## Abstract

**Background:**

Regional citrate anticoagulation may cause a negative calcium balance, systemic hypocalcemia and parathormone (PTH) activation but randomzed studies are not available. Aim was to determine the effect of citrate dose on calcium (Ca) and magnesium (Mg) balance, PTH and Vitamin D.

**Methods:**

Single center prospective randomized study. Patients, requiring continuous venovenous hemofiltration (CVVH) with citrate, randomized to low dose citrate (2.5 mmol/L) vs. high dose (4.5 mmol/L) for 24 h, targeting post-filter ionized calcium (pfiCa) of 0.325–0.4 mmol/L vs. 0.2–0.275 mmol/L, using the Prismaflex® algorithm with 100% postfilter calcium replacement. Extra physician-ordered Ca and Mg supplementation was performed aiming at systemic iCa > 1.0 mmol/L. Arterial blood, effluent and post-filter aliquots were taken for balance calculations (area under the curve), intact (i), oxidized (ox) and non-oxidized (nox) PTH, 25-hydroxy-Vitamin D (25D) and 1,25-dihydroxy-Vitamin D (1,25D).

**Results:**

35 patients were analyzed, 17 to high, 18 to low citrate. Mean 24-h Ca balance was - 9.72 mmol/d (standard error 1.70) in the high vs − 1.18 mmol/d (se 1.70)) (*p* = 0.002) in the low citrate group and 24-h Mg-balance was − 25.99 (se 2.10) mmol/d vs. -17.63 (se 2.10) mmol/d (*p* = 0.008) respectively. Physician-ordered Ca supplementation, higher in the high citrate group, resulted in a positive Ca-balance in both groups. iPTH, oxPTH or noxPTH were not different between groups. Over 24 h, median PTH decreased from 222 (25th–75th percentile 140–384) to 162 (111–265) pg/ml (*p* = 0.002); oxPTH from 192 (124–353) to 154 pg/ml (87–231), *p* = 0.002. NoxPTH did not change significantly. Mean 25 D (standard deviation), decreased from 36.5 (11.8) to 33.3 (11.2) nmol/l (*p* = 0.003), 1,25D rose from 40.9 pg/ml (30.7) to 43.2 (30.7) pg/ml (*p* = 0.046), without differences between groups.

**Conclusions:**

A higher citrate dose caused a more negative CVVH Ca balance than a lower dose, due to a higher effluent Calcium loss. Physician-ordered Ca supplementation, targeting a systemic iCa > 1.0 mmol/L, higher in the high citrate group, resulted in a positive Ca-balance in both groups. iPTH and oxPTH declined, suggesting decreased oxidative stress, while noxPTH did not change. 25D decreased while 1,25-D rose. Mg balance was negative in both groups, more so in the high citrate group.

**Trial registration:**

ClinicalTrials.gov: NCT02194569. Registered 18 July 2014.

**Supplementary Information:**

The online version contains supplementary material available at 10.1186/s12882-021-02598-2.

## Background

Regional anticoagulation with citrate is recommended as first-line anticoagulation for patients on continuous renal replacement therapy (CRRT) [1]. This method is based on pre-filter administration of citrate, which lowers ionized calcium (iCa) by chelation, thereby inhibiting the clotting cascade within the filter, and on the concomitant replacement of calcium (Ca) via the post-filter circuit or a separate line. Insufficient replacement of the increased loss of Ca as Ca citrate complexes in the effluent may lead to a negative Ca balance, systemic hypocalcemia [2–5] and further activation of parathormone (PTH), which may already be activated due to renal dysfunction. Until now calcium balance during citrate CVVH has not been studied in a randomized way.

In our center, the standard citrate CVVH protocol is initiated at a citrate dose of 3.0 mmol/L, targeting post-filter ionized calcium (pfiCa) of 0.25–0.35 mmol/L. Similar values are utilized in most other centres. In clinical practice, citrate doses may be increased in case of higher pfiCa measurements or in case of repeated filter loss due to clotting. Though there is no specific threshold citrate dose which is deemed to be harmful, reduced metabolism of citrate (for example because of liver failure or poor muscle perfusion) may lead to accumulation with secondary hypocalcemia and metabolic acidosis [6]. Monitoring of the total to ionized calcium ratio (T/iCa) is generally utilized as a marker to detect citrate accumulation.

Even a minor decrease in systemic iCa can trigger PTH release, mobilizing Ca from skeletal stores within minutes [7, 8]. A negative Ca balance during citrate-based CRRT has been observed in three studies [2, 4, 5], and in two a rise in intact (i)PTH has been reported [2, 3]. Although it seems rational to replace the lost amount of Ca, in clinical practice Ca replacement is often adjusted to the systemic iCa concentration, despite the fact that the optimal iCa concentration during critical illness is not known. Critically ill patients often have low iCa concentrations [9]. This may be adaptive and supplementation of Ca to normal values might be harmful [10]. Importantly, critical illness-related oxidative stress leads to the oxidation of PTH. While the non-oxidized form of PTH (noxPTH) is a full agonist of the receptor, oxidized PTH (oxPTH) loses its PTH receptor-stimulating properties [11]. The standard measured iPTH measurements do not discriminate between bio-active noxPTH and oxPTH. Changes in mineral metabolism in AKI are complex [12] and both high phosphate and low vitamin D have been implicated, higher phosphate influencing PTH stimulation through Calcium sequestration. 25D deficiency (< 50 nmol/L) has been associated increased rates of sepsis, AKI and mortality [13, 14], though the underlying mechanism remains unclear.

Citrate-based regional anti-coagulation in CRRT increases Magnesium (Mg) loss as well because citrate also chelates Mg [15].

Aim of this short-term proof of concept study was to determine the effect of citrate dose in continuous venovenous hemofiltration (CVVH) on Ca and Mg balance, the effect of physician-ordered Ca supplementation on Calcium balance and the overall effect on PTH and Vitamin D status.

## Methods

A single center prospective randomized study (NCT 02194569) was performed in the mixed medical-surgical ICU of Oost Limburg Hospital, Genk, Belgium, a large non-University teaching hospital. The study was approved by the local ethical committee and written informed consent was obtained from the patient or legal representative. The study included intensive care patients, receiving continuous venovenous hemofiltration (CVVH) with citrate for AKI stage 2 or 3. According to the local protocol, citrate was the first-line anticoagulant for CRRT, but not in patients with suspected liver failure. Patients not expected to survive the next 24 h and patients already receiving renal replacement therapy at the time of admission to ICU were excluded.

### Study protocol

After obtaining written informed consent, patients were randomized to low dose citrate (2.5 mmol/L blood flow) or high dose citrate (4.5 mmol/L blood flow), targeting a post-filter ionized Calcium (pfiCa) of 1.3–1.6 mg/dL (0.325–0.4 mmol/L) and 0.8–1.1 mg/dL (0.2–0.275 mmol/L) respectively. PfiCa was subsequently titrated by increasing or decreasing citrate dose by steps of 0.5 mmol/L. The study was performed for the first 24 h of CVVH treatment after which the clinician was free to change modality according to the standard treatment protocol in our center. If, for whatever reason, the CVVH session was discontinued within 24 h, the patient was not readmitted to the study and the study was stopped. If the CVVH session was discontinued within 12 h of initiation, the patient was excluded from the study.

### CVVH procedure

Citrate CVVH was performed using the Prismaflex® (Baxter international inc. Deerfield IL, USA; formerly Gambro Lundia AB, Lund, Sweden) and a 1.5m^2^ AN69 HF dialyzer (K_uf_ 37 mL/h/ mmHg). Blood flow rate was set according to body weight (see appendix 1) and CVVH clearance was set for a total 30 ml/kg/h after correction for predilution. In the citrate mode, the algorithm asks for a citrate dose setting which reflects the citrate concentration in the extracorporeal circuit. According to randomization, the citrate doses were chosen as described above. As prefilter citrate solution, Prismocitrate 18/0®, a dilute citrate-containing anticoagulation solution was used containing 18 mmol citrate/L. Post-filter substitution was performed utilizing a calcium-free substitution fluid, PrismOcal B22®, containing 0.75 mmol Mg/L (full composition in appendix 2 and appendix 3 respectively). Prismaflex® compensates the extracorporeal loss of free calcium and calcium bound in calcium-citrate complexes using a standardized closed-loop system for the dosing of calcium post-filter (Ca-chloride 550 mmol/L). Calcium compensation can be set from 5 to 200% but was set at 100% during the study. The algorithm is based on the Prismaflex® CVVH settings, as well as hematocrit and the disposable set used (effective surface area). Changes in settings will change calcium loss via effluent and consequently Prismaflex® will adjust the calcium chloride pump speed to maintain the set calcium compensation. Extra Ca supplementation was permitted at the discretion of the treating physician, our standard of care dictating a systemic iCa of more than 1.0 mmol/L. Results for iCa, measured via the blood gas analyzer, were immediately available to the clinician (see below). Magnesium supplementation was not part of the study citrate protocol and the results were available the next day. Fluid removal rates were targeted according to individual needs whilst keeping these constant where possible throughout the study period. Vascular access was established by ultrasonography-guided placement of a 12 F haemodialysis catheter in either jugular, femoral or subclavian vein (in order of preference).

### Study measurements and calculations

Arterial blood was taken at 0, 2, 4, 6, 12, 18 and 24 h to measure total Calcium (tCa) and ionized Ca (iCa) and total Magnesium (tMg). T/iCa, the ratio of total Calcium (tCa) and ionized Ca (iCa), was calculated at each time point. iCa was also measured in postfilter blood (pfiCa). A blood gas analyzer (Radiometer, Copenhagen, Denmark) was used for the measurement of iCa. Post-filter citrate concentrations were measured at 1 and 24 h or at the time of protocol exit, using the following assay:

Citrate Reagent Set (Enzymatic Method, ref. 2881), produced by Firma InstruChemie BV, Delfzijl, The Netherlands. Controls: Citrate/Oxalate control (Normal & High): ref.3085. Parathormone was measured in plasma at 0 and 24 h using an PTH intact assay (Roche Diagnostics (Mannheim, Germany), in an external laboratory (Immundiagnostik AG, Heidelberg, Germany) for measurement of intact iPTH and noxPTH separately. The non-oxidized form of PTH (noxPTH) was measured by removal of the oxidized form (oxPTH), using specific monoclonal antibodies (antihuman oxidized PTH monoclonal antibody (Immundiagnostik AG). OxPTH was calculated by subtracting noxPTH from iPTH.

Both 25-hydroxy-vitamin D and 1,25-dihydroxy-vitamin D were measured at 0 and 24 h, using the following assays: 25 OH Vitamin D: Electrochemiluminescence binding assay (Elecsys Vitamin D total II on Cobas e801; Roche Diagnostics; Rotkreuz, Switzerland). 1.25 OH vitamin D: Automated streptavidin-biotin based immunoassay after immunoextraction (IDS-iSYS1.25 VitD^Xp^ assay; IDS; Newcastle, UK).

All assays were performed according to manufacturers’ instructions, with internal quality controls and participation to external quality control programs.

Phosphate was measured at 0 and 24 h.

Effluent aliquots were taken at 2, 4, 6, 12, 18 and 24 h to measure total calcium and total magnesium (tMg) and CVVH calcium and magnesium loss were calculated (mmol/kg/h) at the different sample times. After calculating calcium and magnesium compensation given via compensation and substitution fluids respectively, it was possible to calculate CVVH balances for calcium and magnesium. Furthermore, these balances were calculated accounting for separate physician-ordered iv calcium and magnesium supplementation. The calcium and magnesium as administered via nutrition was not accounted for. 24-h balances (mmol/kg/d) were calculated as area under the curve (AUC), based on these individual calculations. Formulas for balance calculations are shown in Appendix 4. For readability, balance results are presented after correction for a body weight of 80 kg.

### Data collection

Baseline demographics including sex, age, weight and APACHE II and AKI/KDIGO criteria were collected.

#### Statistical analysis

Analyses were conducted using SPSS version 24.0 statistical software (IBM SPSS statistics) and R, version 3.6.0. Continuous data are presented as mean (standard deviation, SD or SEM) or median (25th–75th percentile); categorical variables are given as numbers and percentages (%). At baseline (0 h) the low and high citrate group are compared in terms of baseline characteristics and concentrations. To compare categorical data the Pearson Chi-square or a Fisher’s exact test were used. For continuous data a two-sample t-test or a Mann-Whitney U test, where appropriate, were used. Normality was tested using the Shapiro–Wilks test. Correlations were calculated using Pearson’s coefficient of correlation or Spearman’s coefficient of correlation, depending on normality. Paired variables were compared using paired samples T-test or Wilcoxon signed rank test where appropriate. Outcomes measured repeatedly over time (obtained from 2 h after initiation of the study onwards) were compared between groups by using linear mixed models (LMMs). The LMM included as fixed effects citrate group, time and their interaction and a random patient effect. The LMM was also used to test for differences by time point. In case a by time point analysis is reported, correction for multiple comparisons was done separately for each endpoint. AUC’s were used to calculate 24 h-calcium and magnesium balance. AUCs were compared between groups and to zero, by means of a t-test.

A p–value of < 0.05 was deemed to be statistically significant. Sample size calculation was based on a power of 80% to compare the mean AUC between the citrate groups by means of a t-test. The sample size was calculated using the initially observed variability and differences in the AUC data in pilot data. The minimum sample size was 15 patients per group for Mg balance and 11 per group for the Ca balance.

## Results

### Patients

**Fig. 1:**
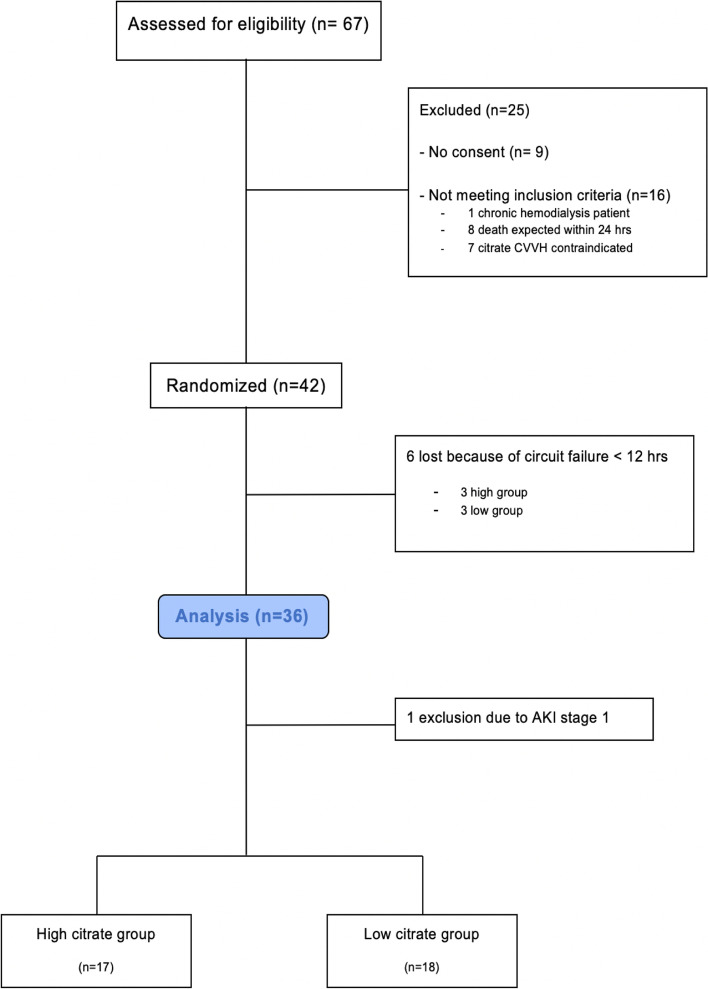
Study enrolment

Sixty-seven patients were assessed for eligibility and 42 patients were randomized for inclusion. Figure 1 describes study enrolment in detail. Of the 42 randomized patients, 35 were included in the statistical analyses, 17 in the high citrate group and 18 in the low citrate group. Six patients had a circuit failure within 12 h and 1 patient was excluded due to AKI stage 1. Thirty three out of 35 patients (94%) completed the entire study period, two patients, both from the low citrate group, were only included until t = 18 because of circuit loss (catheter dysfunction at 20 h and filter coagulation at 22 h).

### Baseline characteristics

Baseline characteristics of the patients (Table 1) and baseline concentrations of total and ionized calcium, magnesium and albumin (Table 2) were not significantly different between the high and low citrate groups.

**Table 1:** Baseline Characteristics

	total	High citrate	Low citrate	*p*-value
no. of patients	35	17	18	
Age	65.4 (14.8)	62.4 (18.5)	68.2 (9.9)	0.267
sex (male)	24 (69%)	10 (59%)	14 (78%)	0.200
Weight (kg)	87.1 (16.7)	85.9 (17.3)	88.3 (16.5)	0.671
AKI stage 2/3	15 (43%)/20(57%)	5 (29%)/12(71%)	10 (56%)/8 (44%)	0.118
APACHE II	30.0 (5.4)	28.5 (5.4)	31.5 (5.0)	0.095
SOFA	12.7 (3.3)	11.88 (3.7)	13.39 (2.7)	0.178
Mechanical ventilation	30 (86%)	13 (76%)	17 (94%)	0.151
Vasopressor/inotropics	23 (66%)	9 (53%)	14 (78%)	0.117
Blood flow (ml/min)	137 (17)	136 (17)	138 (17)	0.671

**Table 2:**
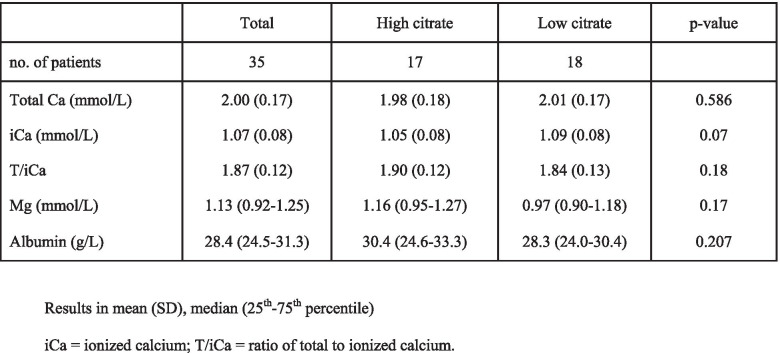
Baseline concentrations of calcium, magnesium and albumin

### Intervention

#### Citrate dose and pfiCa

**Fig. 2:**
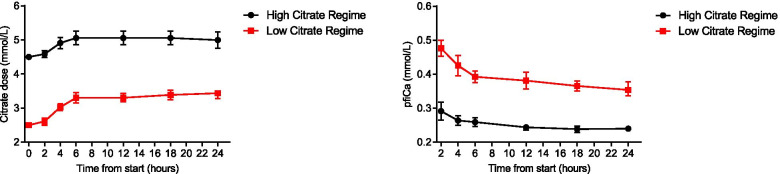
Citrate dose and pfiCa per group

During the study period, citrate dose was adjusted to measured pfiCa values. The initial citrate dose was maintained in 3 of 35 (9%) patients, all in the high citrate group. In all other patients, the citrate dose was increased within the first 4 h. Over the course of 24 h mean citrate dose was 4.88 (se 0.04) mmol/L in the high versus 3.08 (se 0.04) mmol/L in the low citrate group (*p* < 0.0001). Fig. 2 displays the evolution in citrate dose and pfiCa. Based on LMM calculations mean pfiCa was 0.26 (se 0.0038) in the high vs. 0.40 (se 0.0055) mmol/L in the low citrate group (p < 0.0001). CRRT clearance, (calculated based on effluent + fluid removal) (mean high 26.35 ml/kg/h (se 0.17), mean low 26.45 ml/kg/h (se 0.17), *p* = 0.68), did not differ significantly between groups.

#### Systemic Ca and mg concentrations

**Fig. 3:**
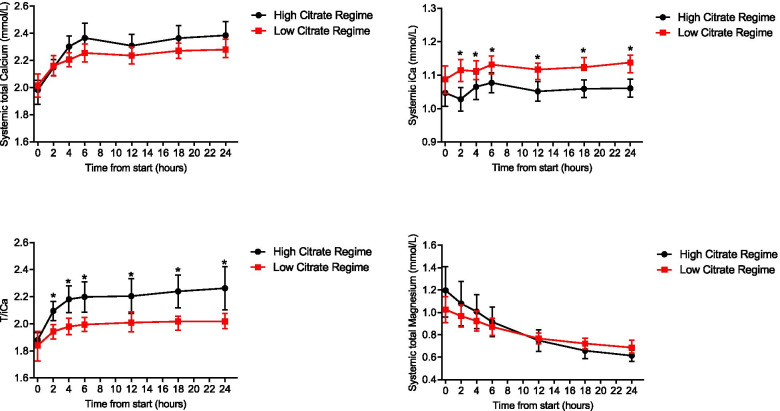
Comparison in High vs Low groups for systemic Total and ionized Calcium, T/I Ca and total systemic Magnesium

The average course of the systemic calcium and magnesium levels is illustrated in Fig. 3. The difference between groups for iCa was constant over the intervention period (group by time interaction *p* = 0.66) and 0.06 mmol/L lower in the high citrate group (*p* < 0.001). Changes over time were the same for both groups (from baseline to 24 h the iCa increased by 0.023 mmol/L, *p* = 0.024).

The pattern of total Ca was comparible between groups (Fig. 3). Total Ca increased until T6. The increase was stronger in the high citrate group (0.065 mmol/L/h versus 0.038 mmol/L/h, *p* = 0.0008). From T6 onwards, the treatment effect was constant and borderline not statistically significant (total Ca for the high citrate group was 0.098 mmol/L higher than for the low citrate group, *p* = 0.052).

At all timepoints T/iCa was signficantly higher in the high than the low citrate group (Fig. 3). The increase until T6 was stronger in the high citrate group (0.051 versus 0.026 per hour, *p* = 0.0033). From T6 onwards, the treatment effect was constant (T/iCa for the high citrate group was on average 0.219 higher than for the low citrate group, *p* = 0.0012).

Total Mg decreased in both groups, but faster during the high than in the low citrate group (− 0.024 mmol/L/h versus − 0.014 mmol/L/h, *p* < 0.0001). Differences per timepoint were not statistically significant (see Fig.3). At baseline 20/35 (57.1%) Mg levels were above normal (normal values 0.7–1.0 mmol/l) and 3 below normal (9%). At 24 h 22/35 (63%) were below 0.7 mmol/l, none were above normal.

#### Post filter citrate concentration, citrate dose and T/iCa

Citrate concentrations were significantly different between groups, both at 1 h (median high was 790 mg/l (715–879) vs. low 482 mg/l (422–567), *p* < 0.001) and at 24 h (median high 925 mg/l (858–1057) vs low 670 mg/l (571–725), p < 0.001). There was a positive correlation between citrate dose and post filter citrate concentration at 1 h (r = 0.776 p < 0.001) and at 24 h (r = 0.864, *p* < 0.01). T/iCa correlated with citrate concentration at 24 h (r = 0.643 *p* < 0.001).

#### CVVH Ca and Mg balances

**Fig. 4:**
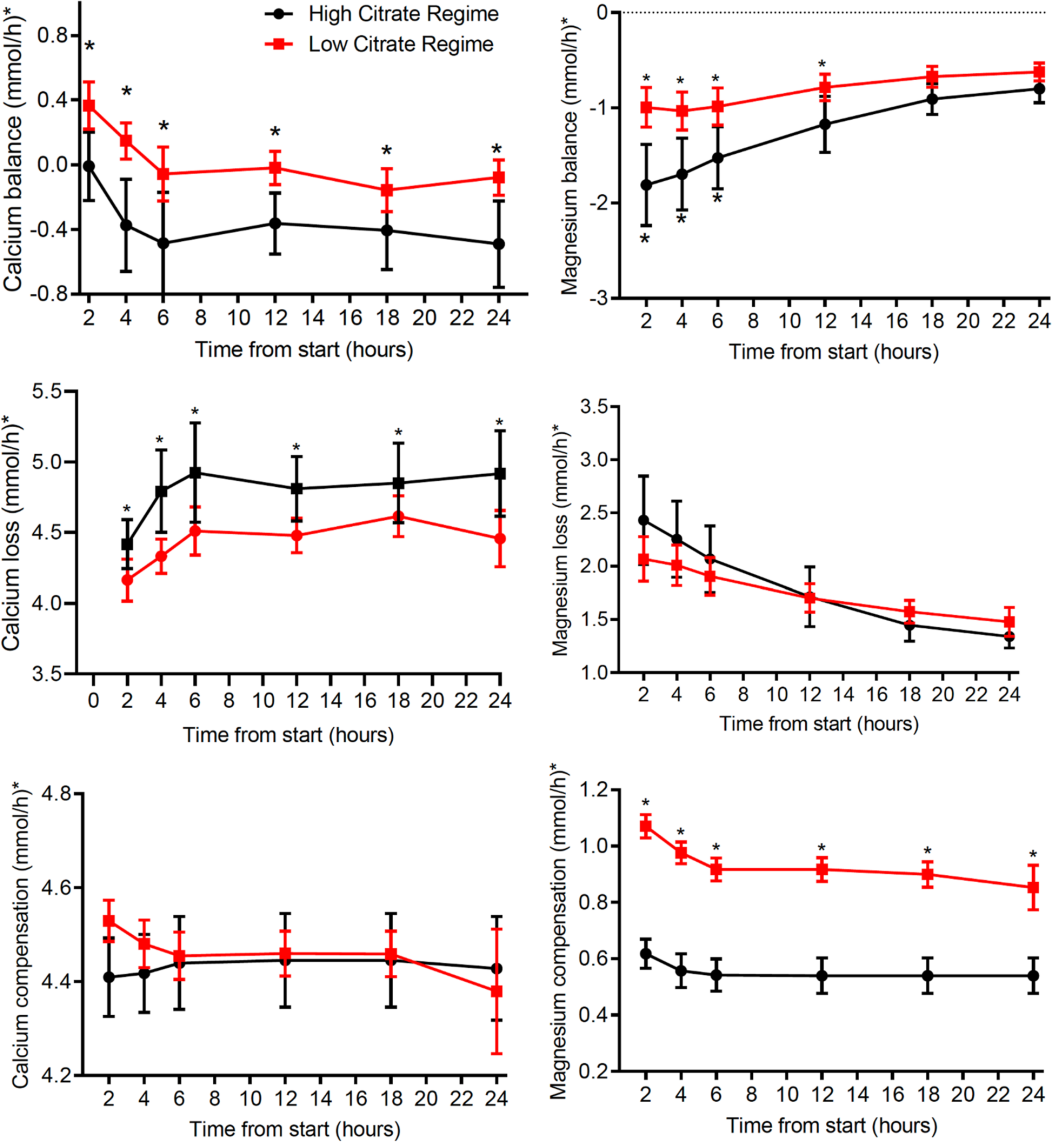
Calcium and Magnesium balances, loss and replacement (*calculated for a person of 80 kg)

For readability, balance results are presented corrected for a body weight of 80 kg. Ca and Mg balances at the different sampling times for the high and the low citrate groups are presented in Fig. 4. For Ca balance the difference between the high and low citrate group was constant over the intervention period (group by time interaction *p* = 0.49).

Ca-balance was more negative in the high than in the low citrate group (difference 0.40 mmol/h (se = 0.1028, *p* = 0.0004)). Ca balance decreased by 0.112 mmol/h between T2-T6 in both groups and stabilizing at − 0.46 mmol/h (se 0.079) for the high citrate group and − 0.074 (se 0.077) mmol/h for the low citrate group. Overall, from T6 onwards, Ca balance was significantly lower than zero in the high group but not in the low group. In the high group, 82 of 101 (81%) of measured Ca balances were negative, while in the low group this was the case in 52 of 107 (49%).

For calcium loss the difference between the high and low citrate group was constant over the intervention period (group by time interaction, *p* = 0.378). Ca-loss was higher in the high than in the low Ca group (difference was 0.37 mmol/h (se = 0.12, *p* = 0.0036)). Ca loss increased by 0.11 mmol/hour between T2-T6 (in both groups) and after that it stabilized at 4.89 (se 0.096) mmol/h for the high citrate group and 4.51 (se 0.093) mmol/h for the low citrate group.

For Ca compensation differences between the 2 groups were not constant over time (group by time interaction p = 0.003). However, after correction for multiple testing, none of the individual time points showed a significant difference between the 2 groups.

The 24-h Ca balance, based on AUC values, was significantly different for the low and high group (*p* = 0.002), being lower than zero in the high citrate group (− 9.27 mmol/d, (se 1.70), *p* < 0.001) but not in the low group (− 1.18 mmol/d, (se 1.70), *p* = 0.49). After taking physician-ordered Ca supplements into account, Ca balance was positive in both groups and more so in the high dose group (*p* = 0.048) (see Table 3). Seventy percent of patients in the high group received Ca supplementation vs. 31% in the low group (*p* = 0.006).

**Table 3:**
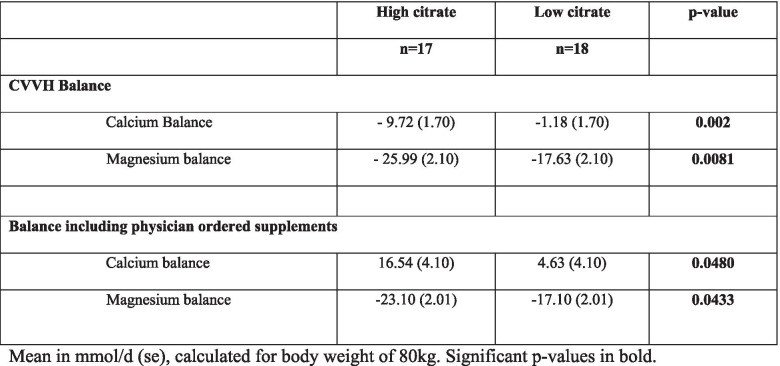
Overall Calcium and Magnesium balances over 24 h

All values for Mg balance were negative irrespective of citrate group and time. The group by time interaction for Mg balance is statistically significant (p < 0.001), indicating that the treatment effect changed over the study period. At T2, T4, T6 and T12, Mg balances where more negative in the high group compared to the low citrate group (all *p* < 0.05) Thereafter, differences were no longer statistically significant.

For Mg loss differences between the 2 groups were not constant over time (group by time interaction *p* < 0.0001). However, after correction for multiple testing, the differences between the 2 groups at individual time points were not significant.

For Mg compensation (via the CRRT fluids) the difference between the high and low citrate group was not constant over the intervention period (group by time interaction p < 0.0001). Mean difference in Mg compensation between the high and the low groups equaled 0.38 mmol/h (se = 0.032, p < 0.0001) with more Mg compensation in the low group. Mg compensation decreased by 0.029 mmol/h between T2-T6 in both groups and then stabilized at 0.54 (se 0.025) mmol/h for the high citrate group and 0.90 (se 0.024) mmol/h for the low citrate group.

The 24-h Mg balance was − 25.99 (2.10) mmol/d in the high group vs. -17.63 (se 2.10) in the low group, both significantly different from zero (*p* < 0.001) and different from each other (*p* = 0.0081) (see Table 3). When accounting for extra supplementation the difference remained significant (*p* = 0.0433) (see Table 3). No statistical difference in amount of extra Mg supplementation could be demonstrated. Almost all Mg supplementation was given at 24 h and was accounted for in the balances revised for extra supplementation.

### iPTH, oxidized and non-oxidized PTH

Baseline iPTH, median 222 (140–384) pg/ml, decreased to 162 (11–265) at 24 h (*p* = 0.002). There were no significant differences between citrate groups both at baseline or at 24 h. The decrease in iPTH was significant in both the high citrate group and the low citrate group (see Table 4). OxPTH decreased from a median of 192 (124–353) pg/ml to 154 (86–231) (p = 0.002), while overall noxPTH at 24 h was not significantly different (see Table 4) compared to baseline (*p* = 0.339). Findings were similar for both high and low groups.

**Table 4:**
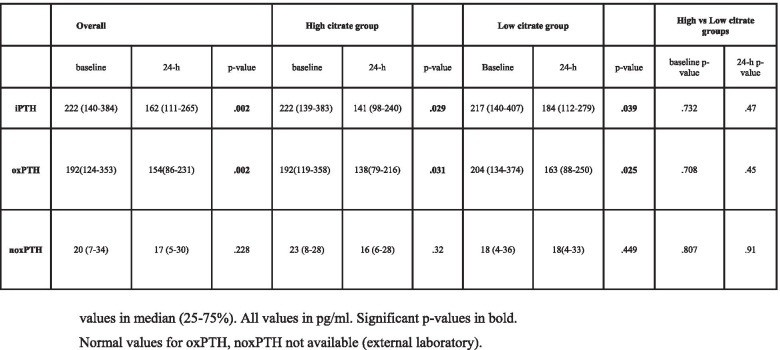
PTH measurements

#### Phosphate

Mean baseline serum Phosphate was 1.85 (0.64) mmol/L and did not differ between groups. After 24 h of citrate CVVH, Phosphate had fallen to 1.05 (0.42) mmol/l (*p* < 0.001). There was no significant difference between groups. At t0 23/35 (66%) were above normal, only 1 patient below normal (normal values 0.9–1.5 mmol/l), at t24 only 2 were above 1.5 mmol/l (7%), 14/35 (40%) were below normal.

#### 25D and 1,25 D

Mean 25 D was 36.5 (11.8) nmol/l before CVVH, dropping minimally, though significantly to 33.3 (11.2) nmol/l after 24 h, a mean drop of 10.2% (*p* = 0.003). At t0 9/35 (25.7%) of values were below 30 nmol/l, 32/35 (91.4%) below 50 nmol/l. After 24 h of CVVH, 11/35 (37.1%) were below 30 nmol/l), 33/35 (94.3%) below 50 nmol/l. Both at t0 and t24 there were no significant differences between groups. Mean 1,25D was 40.9 pg/ml (30.7) before CVVH, rising minimally, though significantly to 43.2 (30.7) pg/ml after 24 h, a mean rise of 11.5% (*p* = 0.046). There were no significant differences between groups, both at t0 and t24.

## Discussion

This is the first randomized controlled trial in critically ill patients comparing calcium and magnesium balance between low and high dose citrate for CVVH. It shows that calcium balance depends on citrate dose. The CRRT calcium balance was more negative in the higher citrate dose group despite setting calcium compensation at 100% in both groups. This was the consequence of higher Calcium loss via effluent in the high citrate group. The CRRT magnesium balance was negative at all time points in both citrate groups, but more negative in the high citrate group. We also found that PTH was increased, both at baseline and at 24-h. In both groups PTH declined during the first 24 h of CVVH. Remarkably, the biologically active noxPTH did not change significantly in time. However, the pattern of oxPTH was similar to that of PTH, suggesting that the decline in PTH may be attributed to a decline in the biologically non-active oxPTH, e.g. to a decline in oxidative stress. More than 90% of patients were Vitamin D deficient at inclusion (25D < 50 nmol/l), and 25D decreased significantly during the study. The clinical implications of these limited falls remain unclear. Conversely 1,25D rose during the study.

Our findings of a negative CRRT calcium balance during citrate-based CRRT (in the high citrate group), are in correspondence with the literature [2, 4, 5]. Van der Voort et al. reported a negative Ca balance while targeting citrate at 2.92 mmol/l and systemic iCa at 0.8–1.0 mmol/l), both lower than our two groups [2]. Similarly, Brain et al. [4] reported a negative Ca balance while targeting systemic iCa at 0.8–1.1 mmol/l; mean citrate concentration was 2.42 mmol/L, lower than in our low group (mean 3.08 mmol/L). Both our citrate dose groups were associated with a higher loss of calcium from the circuit than in the Brain study (4.01 mmol/h), which concurs with the higher circuit citrate levels. The Prismaflex calcium replacement algorithm did a better job of compensating for calcium loss in our low group, resulting in neutral balance, compared to the calcium replacement protocol in the Brain study, which was retrospective and adapted systemic iCa to a lower level. In contrast, in our higher group the Prismaflex algorithm fell short. The negative Prismaflex balance was however compensated by extra physician ordered Ca supplementation, aiming at a systemic iCa concentration of 1.0 mmol/L. Altogether, this is the first study to prove in a randomized controlled setting that a higher citrate dose causes more loss from the circuit, as intuitively expected. It also shows that the calcium balance depends on subsequent calcium replacement, which in turn depends on target systemic iCa.

In both groups, calcium balance became steadily more negative during the first 6 h of CVVH, as calcium loss increased, both stabilizing thereafter. After the start of citrate CRRT, systemic citrate concentration gradually increases until plasma citrate concentration reaches a steady state between citrate administration, loss and metabolism. Citrate-bound calcium increases during the first hours of CRRT until the plateau has been reached, while at the same time Calcium loss via the filter increases. The Zheng study [5] neatly describes this phenomenon. As systemic citrate concentrations gradually rise before leveling off, calcium supplementation in the first 3 h should be higher. A two-phase calcium supplementation protocol has therefore been suggested. In our study this primary phase of increasing Calcium loss before plateau lasted longer, likely because of simultaneous up-titration of citrate target to achieve protocol post filter iCa values, (as illustrated in Fig. 1).

Our study evaluated iPTH, as well as oxPTH and noxPTH, over the first 24 h of citrate CVVH. Median baseline iPTH in our study was lower than the 264 pg/ml reported in the Van der Voort study [2], but higher than the 67 pg/ml in the study by Raimundo et al. [3]. At 24 h, median iPTH in our study dropped, while it rose to 399 pg/ml in the van der Voort study and was 89 pg/ml in the Raimundo study. The differences in PTH values, particularly the lower values in the Raimundo study, could be the consequence of differences in serum iCa values, which were consistently higher in the Raimundo study, though between-assay differences cannot be ruled out. However, our study provides evidence that another factor may explain changes in PTH. The fact that oxPTH drops during citrate CVVH may be due to reduction in inflammation/oxidative stress [11], an effect ascribed to citrate [6] or to metabolic improvement. Remarkably, the biologically active noxPTH remained unchanged. Knowledge of the metabolism of oxPTH in the clinical setting is limited and most studies having taken place over longer periods, for example in chronic HD populations or by comparing values for oxidation of PTH in different populations [16]. Worse outcomes found in patients with higher oxPTH values have been attributed to the effects of higher oxidative stress [17]. To our knowledge this is the first study describing oxPTH in the setting of AKI and (citrate) CVVH. Not only is oxPTH biologically inactive (in contrast to noxPTH), but in an animal model it has also been demonstrated to have a slower metabolic clearance rate than noxPTH [16]. A number of studies have taken place to study how best to shield PTH analogues from oxidation and, amongst others, EDTA, a chelating agent which prevents oxidation by sequestering metal ions which catalyze oxidation, has been shown to reduce rates of oxidation of PTH [18]. Similar properties, but not in the setting of PTH, have been ascribed to citrate [6]. It can be speculated that citrate may limit the one-way oxidation of noxPTH to oxPTH, at least to a certain extent, and that oxPTH which is metabolized more slowly than noxPTH will be replenished less fast, leading to diminishing values over 24 h [6, 16]. However, other factors such as vitamin D status and supplementation as well as phosphate and HDL concentrations have been implicated in changes in noxPTH values and its oxidation, though, as yet, only in non-AKI patients [19].

At inclusion, more than 90% of patients were Vitamin D deficient and a quarter severely so, similar to findings in ICU populations elsewhere [20]. Though 25D fell further and 1,25D conversely rose significantly, clinically these changes seem minor and did not differ between study groups. Knowledge of the Vitamin D levels in AKI in ICU patients, both with or without RRT remains limited [12] and prospective studies may shed further light [21]. As expected, phosphate fell significantly over 24 h, reflecting the effects of renal replacement therapy.

In critically ill patients, hypocalcemia and increased levels of intact serum PTH are common early findings [22]. A Cochrane database review studying the effects of parenteral supplementation of calcium for hypocalcaemia found no clear evidence that calcium supplementation to normal systemic iCa values impacts the outcome of critically ill patients [23]. However, bone resorption due to prolonged immobilization leads to hypercalcemia, usually after 4 weeks [24]. Ca compensation based on measured systemic iCa, can thus either mask or exacerbate these underlying trends in calcium flux. Wang et al. [25] provided anecdotal evidence of the former, leading to catastrophic bone loss and spontaneous fractures after citrate-based CRRT for 120 days. The Prismaflex calcium replacement algorithm if set at 100% and used at lower citrate targets can safeguard against the consequences of underlying calcium flux. The algorithm was designed with a citrate target of 3.0 mmol/L in mind and not designed for higher citrate values (personal communication, Mr. Dominique Pouchoulin, Baxter international formerly Gambro). For higher citrate doses, the algorithm needs to be adjusted.

Compared to heparin anticoagulation, renal replacement with citrate-based regional anti-coagulation increases Mg loss because chelation by citrate increases effluent loss [15, 26]. Post-filter substitution fluid contained 0.75 mmol/L of Magnesium so that, depending on the amount of post-filter substitution given, Magnesium loss was partially replaced. However, when increasing citrate dose (and consequently pre-filter volume) the post filter substitution volume was simultaneously lowered to maintain clearance at 30 ml/kg/h. As a result, when using the Prismaflex method, magnesium supplementation unintentionally decreases when citrate dose is increased. Consequently, higher citrate doses not only imply more Mg chelation and loss, but also lower Mg replacement post filter. As suggested before, a negative Mg balance can be alleviated, by using a post filter fluid with a higher Mg concentration (1.5 mmol/L) [27], by using more concentrated citrate solutions pre-filter, and by increasing systemic Mg supplementation based on more frequent controls, e.g. every 6 h.

### Strengths and limitations

This is the first randomized controlled study presenting the effects of high vs. low citrate concentrations on calcium balance whilst using an algorithm delivering 100% calcium compensation and evaluates PTH and Vitamin D using this algorithm, though only over the first 24 h of citrate CVVH. Furthermore, it is the first study measuring oxPTH in the setting of citrate CVVH allowing the calculation of noxPTH, which may reflect the hormone status more precisely. There are also limitations to this study. This study was single center and included only a small number of patients. Citrate doses were chosen to maximize the differences in calcium and magnesium loss and possible effects on PTH and vitamin D metabolism, though the dose in the higher group would not normally be applied clinically for longer periods. However, in clinical practice citrate doses may be increased and approach the higher dose regimen in case of higher PfiCa measurements or in case of repeated filter loss due to clotting.

This short-term mechanistic study was not designed to evaluate long-term effects of citrate on bone metabolism, though it should raise awareness for the possible pitfalls in long term use of citrate CVVH, especially in patients displaying hypercalcemia after prolongend immobiization. The study was also not designed to study the effects of citrate dose on filter lifespan, as clinicians were free to return to standard clinical practice for citrate CVVH after the 24 h of the study and differences between groups on citrate dose were therefore lost. Extra calcium, magnesium and phosphate supplementation (apart from the post-filter substitution fluid) was left to the discretion of the treating ICU physician, although the calcium target was protocolized. Ionized magnesium was not measured as part of the study. Effects of fecal loss, urinary loss or enteral feeding on calcium and magnesium balance were not taken into account. Post-filter iCa was measured using the ABL800 Flex blood gas analyzer (Radiometer, Copenhagen, Denmark). This habit is common, though the reliability of measuring iCa in this low range has been challenged [28].

## Conclusions

This randomized controlled trial in critically ill patients on citrate CVVH is the first to demonstrate that calcium loss depends on citrate dose. Physician-ordered Ca supplementation, targeting a systemic iCa > 1.0 mmol/L higher in the high citrate group, prevented a negative Ca balance. iPTH was increased, both at baseline and at 24-h but declined during the first 24 h of citrate CVVH. According to the literature, this decline in iPTH seems to be related to the systemic iCa target used. However, we found that the pattern of oxPTH was similar to that of iPTH, suggesting that the decline in PTH during CVVH may be attributed to a decline in oxidative stress, while biologically active noxPTH did not change significantly. In the light of our findings the use of iPTH solely, when studying patients with AKI, seems of limited value as this mainly reflects oxidative stress. Further studies measuring oxPTH and noxPTH in this context should prove of value. Undoubtedly, ongoing studies will further elucidate vitamin D metabolism in AKI patients on ICU. Mg balance was negative at all time points in both citrate groups, but more negative in the high citrate group supporting previous findings that the present Mg concentration in the replacement fluids is too low. Mg replacement based on frequent Mg sampling should become standard care.

## Supplementary Information

**Additional file 1.**

## Data Availability

The datasets during and/or analysed during the current study available from the corresponding author on reasonable request.

## Abbreviations

AKI: Acute kidney injury
Ca: Calcium
iCa: Ionized Calcium
pfiCa: Post-filter ionized Calcium
T/iCa: Total to ionized calcium ratio
CVVH: Continuous venovenous hemofiltration
CRRT: Continuous renal replacement therapy
25 D: 25-hydroxy-vitamin D
1,25 D: 1,25-dihydroxy-vitamin D
Mg: Magnesium
PTH: Parathormone
oxPTH: Oxidized Parathormone
noxPTH: Non-oxidized Parathormone
LMM: Linear mixed model
