# Supplementary material for: Citrate dose for continuous hemofiltration: effect on calcium and magnesium balance, parathormone and vitamin D status, a randomized controlled trial
Source: BMC Nephrol. 2021 Dec 11;22:409. doi: 10.1186/s12882-021-02598-2 (PMC8665615; doi:10.1186/s12882-021-02598-2)
Supplement: Supplementary file 1 — Additional file 1. [file 12882_2021_2598_MOESM1_ESM.docx]

**Appendix**

**Appendix 1. Blood flow and bodyweight**

| **Body weight (kg)** | **Blood flow (ml/min)** |
| --- | --- |
|  |  |
| 50 | 100 |
| 60 | 110 |
| 70 | 120 |
| 80 | 130 |
| 90 | 140 |
| 100 | 150 |
| 110 | 160 |
| 120 | 170 |
| 130 | 180 |

**Appendix 2. Prismocitrate18/0**

| **Prismocitrate 18/0** | **mmol/L** |
| --- | --- |
| Citrate | 18 |
| Sodium | 140 |
| Chloride | 86 |

**Appendix 3. Prism0cal B 22**

**Appendix 4. Formulas for balance calculations**

***CVVH Ca balance (mmol/h) = ([Ca]_infusion_ x Q_infusion_)- ([Ca]_eff_ x Q_eff_)***

*[Ca]infusion = Calcium concentration of Ca substitution fluid*

*Qinfusion = volmetric flow rate (L/h) of Ca substitution pump*

*[Ca]eff = Calcium concentration of effluent*

*Qeff = volumetric flow rate (L/h) of effluent**

***CVVH Mg balance (mmol/h) = ([Mg]_replacement_ x Q_replacement_) – ([Mg]_effl_ x Q_effl_)***

*[Mg]replacement = Magnesium concentration in substitution fluid*

*Qreplacement = volumetric flow rate (L/h) of substitution fluid*

*[Mg]effl = Magnesium concentration of effluent*

*Qeff = volumetric flow rate (L/h) of effluent**

**volumetric flow rate of effluent = volumetric flow rate (L/h) of substitution fluid + volumetric flow rate (L/h) of citrate pump + fluid removal rate (L/h)*
